# Interacting and joint effects of triglyceride-glucose index (TyG) and body mass index on stroke risk and the mediating role of TyG in middle-aged and older Chinese adults: a nationwide prospective cohort study

**DOI:** 10.1186/s12933-024-02122-4

**Published:** 2024-01-13

**Authors:** Rong-Rui Huo, Qian Liao, Lu Zhai, Xue-Mei You, Yan-Li Zuo

**Affiliations:** 1 Department of Experimental Research, Guangxi Medical University Cancer Hospital, Nanning, China; 2 Department of Epidemiology and Biostatistics, School of Public Health, Guangxi Medical University, Shuang Yong Rd. #22, Nanning, 530021 China; 3 Department of Smart Health Elderly Care Services and Management, School of Nursing, Guangxi Health Science College, Nanning, China; 4 Key Laboratory of Early Prevention and Treatment for Regional High Frequency Tumour (Guangxi Medical University), Ministry of Education, Nanning, China; 5 Guangxi Key Laboratory of Early Prevention and Treatment for Regional High Frequency Tumour, Nanning, China

**Keywords:** Stroke, Triglyceride glucose index, Body mass index, Mediating effect, CHARLS

## Abstract

**Background:**

Individuals who are overweight or obese often develop insulin resistance, mediation of the association between body mass index (BMI) and stroke risk through the triglyceride-glucose index (TyG) seems plausible but has not been investigated. This study aims to examine whether TyG mediates associations of BMI with stroke risk and the extent of interaction or joint relations of TyG and BMI with stroke outcome.

**Methods:**

The China Health and Retirement Longitudinal Study, initiated in 2011, is a nationally representative, ongoing prospective cohort study involving 8 231 middle-aged and older Chinese adults without a stroke history at baseline. Exposures examined include BMI and the TyG, the latter being the logarithmized product of fasting triglyceride and glucose concentrations. The primary study outcome is stroke incidence, as determined through self-reports, with a follow-up period extending from June 1, 2011, to June 30, 2018.

**Results:**

Of the 8 231 participants, 3 815 (46.3%) were men; mean (SD) age was 59.23 (9.32) years. During a median follow-up of 7.1 years, 585 (7.1%) participants developed stroke. The TyG was found to mediate the association between BMI and incident stroke, proportions mediated were 16.3% for BMI in the 24.0–27.9 kg/m^2^ group and 53.8% for BMI ≥ 28.0 kg/m^2^ group. No significant multiplicative and additive interactions were found between BMI and TyG on incident stroke (Additive: RERI = 1.78, 95% CI − 1.29–4.86; Multiplicative, HR = 1.40, 95% CI 0.86–2.27). HRs for individuals with BMI ≥ 28.0 kg/m^2^ and quartile 4 of TyG compared with those with BMI < 24.0 kg/m^2^ and quartile 1 of TyG were 2.05 (95% CI 1.37–3.06) for incident stroke. Combining BMI and TyG enhanced predictive performance for stroke when compared to their individual (AUC_BMI+TyG_ *vs* AUC_BMI_ *vs* AUC_TyG_, 0.602 *vs* 0.581 *vs* 0.583).

**Conclusions:**

TyG appeared to be associated with stroke risk and mediates more than 50% of the total association between BMI and stroke in middle-aged and older Chinese adults. Public health efforts aiming at the reduction of body weight might decrease the stroke risk due to insulin resistance and the burden of stroke.

**Supplementary Information:**

The online version contains supplementary material available at 10.1186/s12933-024-02122-4.

## Introduction

Stroke remains a leading cause of mortality and long-term disability worldwide, necessitating a deeper understanding of its risk factors for effective prevention and management [1, 2]. Traditionally, body mass index (BMI) has been employed as a principal metric for assessing obesity, which is a well-established risk factor for stroke [3, 4]. However, BMI, a measure based solely on height and weight, has limitations in capturing the complexity of metabolic health. It does not account for muscle mass, fat distribution, overall body composition, and various metabolic markers that are increasingly recognized as significant predictors of cardiovascular risk [5]. As a result, there is a growing interest in identifying more comprehensive markers that can offer additional insights into stroke risk beyond what BMI can provide. In this context, the triglyceride-glucose index (TyG) has emerged as a promising marker [6]. TyG is calculated using fasting levels of triglycerides and glucose and has been proposed as an indicator of insulin resistance [7, 8]. Insulin resistance is a key factor in the pathogenesis of a range of cardiovascular diseases, including stroke [9, 10]. Previous studies [6, 11–13] have shown that TyG is associated with arterial stiffness, coronary artery disease, and even the severity of cerebral infarction, making it a potentially valuable tool in understanding and predicting stroke risk.

Both BMI and TyG are individually associated with stroke [3, 4, 14, 15], in addition, recent studies introduced a novel index called the triglyceride glucose-body mass index (TyG-BMI), and it has also been demonstrated to be associated with stroke [16–18]. However, the interplay between these two metrics remains largely unexplored. Understanding the mediating role of TyG could provide valuable insights into the mechanisms through which BMI influences stroke risk, potentially revealing new avenues for intervention, leading to more targeted interventions that can mitigate this risk by addressing insulin resistance, thereby enhancing stroke prevention and patient care. Additionally, this study aims to explore the extent of interaction or joint relations of TyG and BMI with stroke outcomes. It is crucial to understand whether the combined effect of high BMI and abnormal TyG levels has a synergistic, additive, or antagonistic impact on stroke risk and outcomes. Such knowledge could significantly influence how clinicians assess stroke risk and could lead to more personalized, effective prevention strategies.

This study seeks to fill a critical gap in the existing literature by examining the potential mediating role of TyG in the association between BMI and stroke risk, as well as investigating the combined impact of TyG and BMI on stroke outcomes, drawing data from the "China Health and Retirement Longitudinal Study (CHARLS)", a national cohort study in progress. By offering a more nuanced understanding of the metabolic factors influencing stroke risk, this study aims to contribute to the development of more effective and targeted preventive measures.

## Methods

### Study population

This study conducts a secondary analyses of data from CHARLS, an ongoing nationwide cohort study aimed at representing the population [19]. The study's design has been previously detailed [19]. In brief, it involved the selection of 17 708 participants residing in 10 257 households, employing a multistage stratified probability-proportional-to-size sampling technique. These participants were recruited from 150 counties or districts and 450 villages within 28 provinces in China, covering the period from June 2011 to March 2012. A standardized questionnaire gathered information on sociodemographic and lifestyle factors, along with health-related data. The initial survey (Wave 1) achieved an 80.5% response rate, with subsequent follow-up assessments every two years (Wave 2 in 2013, Wave 3 in 2015, and Wave 4 in 2018). Inclusion criteria for this analyses required participants to be aged 45 years or older, with complete data on fasting blood glucose (FBG), triglycerides (TG), and body mass index (BMI). Individuals with a baseline history of stroke were excluded.

The CHARLS study received approval from the institutional review board at Peking University, and written informed consent was obtained from all participants. This study adhered to the Strengthening the Reporting of Observational Studies in Epidemiology (STROBE) guidelines [20].

### Assessment of BMI and TyG

The Chinese Center for Disease Control and Prevention in Beijing promptly received venous blood samples within two weeks of their departure from the Centers for Disease Control and Prevention (CDC) station. These samples were immediately stored and frozen at -20°C before delivery. After completing necessary assays at the Chinese Medical University laboratory, they were transferred to a deep freezer and maintained at – 80 °C. At the Youanmen Clinical Laboratory of Capital Medical University, TG and FPG concentrations were determined using the enzyme colorimetric assay. The TyG index was calculated as ln[TG (mg/dl) × FPG (mg/dl)/2]. Trained nursing professionals measured height and weight, allowing for the computation of BMI as the weight in kilograms divided by the square of the height in meters.

### Outcome ascertainment

The primary outcome of this study was stroke. Following established precedents [21, 22], stroke occurrences were determined based on self-reports where individuals affirmed having received a diagnosis of stroke from a physician. The date of stroke diagnosis was recorded as falling between the date of the most recent interview and the date of the interview in which the incident stroke was reported [21, 22].

### Covariates

During the initial assessment at Wave 1, trained interviewers gathered sociodemographic and health-related data via a structured questionnaire, including age, gender, residence, marital status, and educational level (categorized as no formal education, primary school, middle or high school, or college and above). Marital status was classified as either married or another marital status (including individuals who were never married, separated, divorced, or widowed). Health-related variables included self-reported smoking and drinking status (categorized as never, former, or current), self-reported physician-diagnosed medical conditions (diabetes, hypertension, heart disease, kidney disease, and dyslipidemia), and the use of medications for diabetes, hypertension, and dyslipidaemia. Laboratory tests included measurements of total cholesterol (TC), high-density lipoprotein cholesterol (HDL-C), low-density lipoprotein cholesterol (LDL-C), estimated glomerular filtration rate (eGFR), and glycosylated hemoglobin, type A1c (HbA1c) [23].

Diabetes was defined as fasting plasma glucose ≥ 126 mg/dL, current use of antidiabetic medication, or self-reported history of diabetes. Hypertension was defined as systolic blood pressure ≥ 140 mmHg, diastolic blood pressure ≥ 90 mmHg, current use of antihypertensive medication, or self-reported history of hypertension. Dyslipidemia was defined as total cholesterol levels ≥ 240 mg/dL, triglyceride levels ≥ 150 mg/dL, low-density lipoprotein cholesterol levels ≥ 160 mg/dL, high-density lipoprotein cholesterol levels < 40 mg/dL, current use of lipid-lowering medication, or self-reported history of dyslipidemia.

### Statistical analyses

Data were described as means and standard deviations (SDs) for normally distributed continuous variables, and as medians and interquartile ranges for nonnormally distributed continuous variables. Frequency with percentage was used to describe categorical variables. Baseline characteristics were compared between groups using X^2^ or analysis of variance or Kruskal–Wallis rank sum test where appropriate. Three percent (275 of 8 231) of total data items were missing, assumed to be missing at random, and, thus, were imputed with the multiple imputation of chained equations method using the baseline characteristics. We created an imputed dataset using the R statistical software version 4.3.0 (R Foundation) along with the mice package.

We used Cox proportional hazard regression models to estimate the hazard ratios (HRs) and 95% confidence intervals (CIs) of outcomes associated with BMI (categorized as < 24.0 kg/m^2^, 24.0–27.9 kg/m^2^, and ≥ 28.0 kg/m^2^) and TyG (categorized as quartiles). Three models were estimated: in model 1, age and sex were adjusted; in model 2, age, sex, residence, marital status, educational level, smoking status, and drinking status were adjusted; and in model 3, the variables in model 2 plus history of diabetes, hypertension, dyslipidemia, and kidney disease; and use of hypertension medications, diabetes medications, and lipid-lowering therapy were adjusted. In addition, we explored the potential nonlinear associations using 3-knotted restricted cubic spline (RCS) regression.

We proceeded to assess indirect associations mediated by TyG and direct associations unmediated by TyG. TyG was dichotomized using the cutoff (9.15) determined by the RCS model. Employing the "mets" package in R, we applied a regression-based approach to compute the total effect, natural indirect effects (NIE), and natural direct effects (NDE) of BMI (< 24.0 kg/m^2^, 24.0–27.9 kg/m^2^, and ≥ 28.0 kg/m^2^) on stroke incidence (Additional file 1: Fig. S1). Two models were constructed: one involved a multivariable logistic regression model for TyG (mediator), conditioned on BMI (exposure) and confounders, while the other encompassed a multivariable Cox proportional hazard regression model for stroke (outcome), conditioned on BMI, TyG, and confounders. NDE conveyed the impact of BMI on stroke independent of TyG, whereas NIE represented the proportion of BMI influenced by its connection with TyG changes over time. To gauge the extent of mediation, we computed the proportion of the association mediated by TyG as NIE divided by the sum of NDE and NIE.

We further conducted a stratified analyses by BMI to investigate the associations of TyG with stroke events among participants in different BMI subgroups. To quantify the additive and multiplicative interactions, we additionally included a product term of BMI (< 24.0 kg/m^2^, 24.0–27.9 kg/m^2^, and ≥ 28.0 kg/m^2^) and TyG (quartiles) in the model. The HR with its 95% CI of the product term was the measure of interaction on the multiplicative scale. We assessed additive interactive effects using three distinct metrics: relative excess risk due to interaction (RERI), proportion attributable to interaction (AP), and synergy index (SI). These metrics capture different aspects of interaction, including the part of the effect attributable to interaction, the proportion of the combined effect arising from interaction, and the ratio between the combined effect and individual effects. Specifically, RERI = 0, AP = 0, and SI = 1 indicate the absence of interactive effects between BMI and TyG concerning stroke incidence. Conversely, when RERI > 0, AP > 0, and SI > 1, this signifies that the combined effects of BMI and TyG on stroke incidence exceed the sum of their individual effects, suggesting synergistic effects. Conversely, if RERI < 0, AP < 0, and SI < 1, it indicates that the combined effects are smaller than the sum of the individual effects of BMI and TyG. We computed the corresponding 95% CIs for these three metrics using the delta method.

To assess the joint associations, we stratified participants into twelve distinct groups based on their BMI (< 24.0 kg/m^2^, 24.0–27.9 kg/m^2^, and ≥ 28.0 kg/m^2^) and TyG (quartiles). Within these groups, we calculated HRs for stroke incidence, comparing them to those individuals with a BMI of < 24.0 kg/m^2^ and TyG falling within quartile 1 as the reference group.

We assessed the performance of BMI, TyG, TyG-BMI index, and TyG + BMI by evaluating their discriminative capabilities using the receiver operating characteristic (ROC) curve and calculating the area under the ROC curve (AUC). Subsequently, we employed the net reclassification index (NRI) and integrated discrimination improvement (IDI) index to further assess the incremental predictive value of BMI + TyG compared to BMI or TyG individually. Finally, the clinical benefits were compared using decision curve analysis (DCA). which plots net benefits against different threshold probabilities [24]. In the decision curve, a reference line called the treat-for-all scheme represents the maximum clinical costs, while a reference line named the treat-for-none scheme indicates no clinical benefit. A decision curve positioned further away from these reference lines suggests a greater clinical value of the prediction variable. Additionally, we also evaluate the incremental predictive value of BMI and TyG index beyond conventional risk factors.

To test the robustness and potential variations in different subgroups, we repeated all analyses stratified by gender (male and female), and age groups (< 60 years, and ≥ 60 years, defined as elders by the World Health Organization [25]). And, sensitivity analyses were conducted as follows: (1) repeating all analyses using the complete data set (7 956 participants) without multiple imputations; (2) using TyG at Wave 3 and excluded participants who have stroke before Wave 3 to minimize the possibility of reverse causality on the observed associations (Of 5 594 participants, 423 participants occured stroke during a median follow-up of 3.0 years); (3) calculating E-values to assess the potential impact of unmeasured confounders on conclusions in observational studies; and (4) using a four-way decomposition method [26] to account for mediation and interaction of BMI and TyG on stroke risk with the CMAverse R package. All analyses were performed using R statistical software version 4.3.0 (R Foundation). We considered two sided *P* values < 0.05 to be significant.

## Results

### Population characteristics

Of the 17 708 CHARLS participants at study baseline, we excluded 777 individuals younger than 45 years, 634 with stroke at baseline, 6 392 without blood samples, 1 655 with incomplete information on BMI, and TyG, and 19 with extreme values on BMI. Finally, 8 231 participants were included for analyses. A detailed description of the selection process for the study analytic sample is included in the Additional file 1: Fig. S2. A comparison of baseline characteristics between participants included and those who were not included in the analysis is shown in Additional file 1: Table S1-2. Excluded participants were more likely to have higher BMI and TyG.

A total of 8 231 adults were included in the analyses. The mean (SD) age at baseline was 59.23 (9.32) years; 3 815 (46.3%) of the participants were men. Table 1 shows the characteristics of the participants according to BMI. At baseline, the mean (SD) TyG was 8.67 (0.66), while the mean (SD) BMI was 23.47 (3.78) kg/m^2^.

**Table 1:** Baseline characteristics participants according to body mass index

Characteristic	Overall (n = 8231)	BMI (kg/m^2^)			*P* value ^a^
		< 24.0 (n = 4882)	24.0–27.9 (n = 2414)	≥ 28.0 (n = 935)	
Age, years	59.23 ± 9.32	60.38 ± 9.62	57.81 ± 8.67	56.88 ± 8.36	< 0.001
< 60	4571 (55.5%)	2495 (51.1%)	1468 (60.8%)	608 (65.0%)	
≥ 60	3660 (44.5%)	2387 (48.9%)	946 (39.2%)	327 (35.0%)	
Gender					< 0.001
Male	3815 (46.3%)	2525 (51.7%)	981 (40.6%)	309 (33.0%)	
Female	4416 (53.7%)	2357 (48.3%)	1433 (59.4%)	626 (67.0%)	
Marital status					< 0.001
Marred	6857 (83.3%)	3955 (81.0%)	2090 (86.6%)	812 (86.8%)	
Other	1374 (16.7%)	927 (19.0%)	324 (13.4%)	123 (13.2%)	
Residence					< 0.001
Urban	2936 (35.7%)	1485 (30.4%)	1015 (42.0%)	436 (46.6%)	
Rural	5295 (64.3%)	3397 (69.6%)	1399 (58.0%)	499 (53.4%)	
Education level					< 0.001
No formal education	2461 (29.9%)	1558 (31.9%)	651 (27.0%)	252 (27.0%)	
Primary school	3345 (40.6%)	2060 (42.2%)	926 (38.4%)	359 (38.4%)	
Middle or high school	2175 (26.4%)	1147 (23.5%)	735 (30.4%)	293 (31.3%)	
College or above	250 (3.0%)	117 (2.4%)	102 (4.2%)	31 (3.3%)	
Smoking status ^b^					< 0.001
Never	5009 (60.9%)	2702 (55.3%)	1619 (67.1%)	688 (73.6%)	
Former	717 (8.7%)	393 (8.0%)	236 (9.8%)	88 (9.4%)	
Current	2483 (30.2%)	1769 (36.2%)	555 (23.0%)	159 (17.0%)	
Drinking status ^b^					< 0.001
Never	4818 (58.5%)	2706 (55.4%)	1495 (61.9%)	617 (66.0%)	
Former	684 (8.3%)	414 (8.5%)	183 (7.6%)	87 (9.3%)	
Current	2725 (33.1%)	1759 (36.0%)	735 (30.4%)	231 (24.7%)	
History of comorbidities					
Hypertension ^b^	2137 (26.0%)	920 (18.8%)	759 (31.4%)	458 (49.0%)	< 0.001
Diabetes ^b^	502 (6.1%)	180 (3.7%)	202 (8.4%)	120 (12.8%)	< 0.001
Heart disease ^b^	965 (11.7%)	471 (9.6%)	317 (13.1%)	177 (18.9%)	< 0.001
Dyslipidemia ^b^	792 (9.6%)	255 (5.2%)	323 (13.4%)	214 (22.9%)	< 0.001
Kidney disease ^b^	483 (5.9%)	286 (5.9%)	142 (5.9%)	55 (5.9%)	> 0.999
History of medication use					
Hypertension medications ^b^	1597 (19.4%)	617 (12.6%)	603 (25.0%)	377 (40.3%)	< 0.001
Diabetes medications ^b^	323 (3.9%)	123 (2.5%)	111 (4.6%)	89 (9.5%)	< 0.001
Dyslipidemia medications ^b^	410 (5.0%)	118 (2.4%)	170 (7.0%)	122 (13.0%)	< 0.001
SBP, mmHg	129.30 ± 21.25	126.75 ± 21.07	131.72 ± 20.78	136.39 ± 21.08	< 0.001
DBP, mmHg	75.19 ± 12.10	73.08 ± 11.69	77.41 ± 11.85	80.49 ± 12.22	< 0.001
TC, mg/dl	194.59 ± 38.77	191.31 ± 37.93	198.60 ± 39.05	201.38 ± 40.56	< 0.001
HDL-C, mg/dl	51.50 ± 15.28	54.92 ± 15.73	47.55 ± 13.30	43.91 ± 12.13	< 0.001
LDL-C, mg/dl ^b^	117.67 ± 34.88	114.89 ± 33.91	121.79 ± 34.99	121.58 ± 38.02	< 0.001
HbA1c, % ^b^	5.29 ± 0.82	5.22 ± 0.76	5.36 ± 0.89	5.50 ± 0.91	< 0.001
Median hsCRP (IQR), mg/l	1.03 (0.55, 2.18)	0.85 (0.49, 1.89)	1.18 (0.64, 2.23)	1.64 (0.89, 3.35)	< 0.001
eGFR, ml/min/1.73m^2 b^	76.86 ± 42.82	76.33 ± 47.03	77.52 ± 35.23	77.94 ± 37.20	0.384
FBG, mg/dl	110.02 ± 35.82	107.12 ± 34.24	113.19 ± 37.92	116.98 ± 36.69	< 0.001
Median TG (IQR), mg/dl	104.43 (74.34, 150.45)	92.04 (67.48, 130.98)	118.59 (84.96, 173.24)	142.49 (100.00, 203.55)	< 0.001
TyG	8.67 ± 0.66	8.53 ± 0.60	8.83 ± 0.67	9.03 ± 0.70	< 0.001
TyG-BMI ^c^	204.22 ± 40.50	179.32 ± 22.76	227.28 ± 20.96	274.66 ± 32.05	< 0.001

Data are presented as mean ± SD or n(%), unless otherwise specified

BMI, body mass index; DBP, diastolic blood pressure; eGFR, estimated glomerular filtration ratio; FBG, fasting blood glucose; HbA1c, glycated hemoglobin; HDL-C, high-density lipoprotein cholesterol; IQR, interquartile range; LDL-C, low-density lipoprotein cholesterol; SD, standard deviation; SBP, systolic blood pressure; TC, total cholesterol; TG, triglyceride; TyG, triglyceride-glucose

^a^ *P* value was based on χ^2^ or analysis of variance or Kruskal–Wallis rank sum test where appropriate

^b^ Missing data: 22 for smoking status, 4 for drinking status, 43 for hypertension, 82 for diabetes, 33 for heart disease, 174 for dyslipidaemia, 43 for kidney disease, 44 for history of medication use for hypertension, 83 for history of medication use for diabetes, 178 for history of medication use for dyslipidaemia, 80 for SBP, 81 for DBP, 16 for LDL-C, 63 for HbA1c, 3 for eGFR

^c^ The TyG-BMI was calculated by the formula ln[TC (mg/dl) × FBG (mg/dl) / 2] × BMI (kg/m^2^)

### Mediation analyses of BMI of TyG with incident stroke

**Fig. 1:**
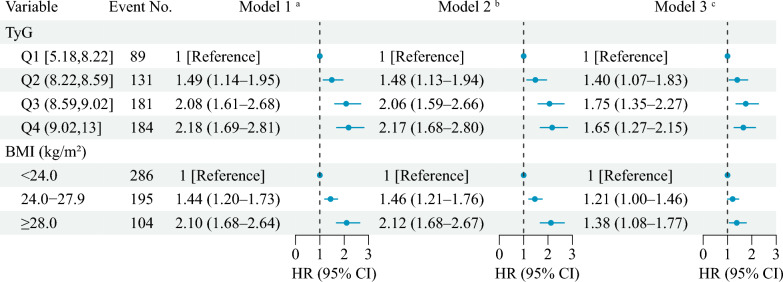
Associations of triglyceride-glucose index and body mass index with stroke. **a** Model 1 was adjusted for age, gender. **b** Model 2 was adjusted for age, gender, marital status, residence, education level, smoking status, and drinking status. **c** Model 3 was adjusted as model 2 plus hypertension, diabetes, heart disease, dyslipidaemia, kidney disease, and history of medication use for hypertension, diabetes, and dyslipidaemia. BMI, body mass index; CI, confidence interval; HR, hazard ratio; Q, quartile; TyG, triglyceride-glucose

**Fig. 2:**
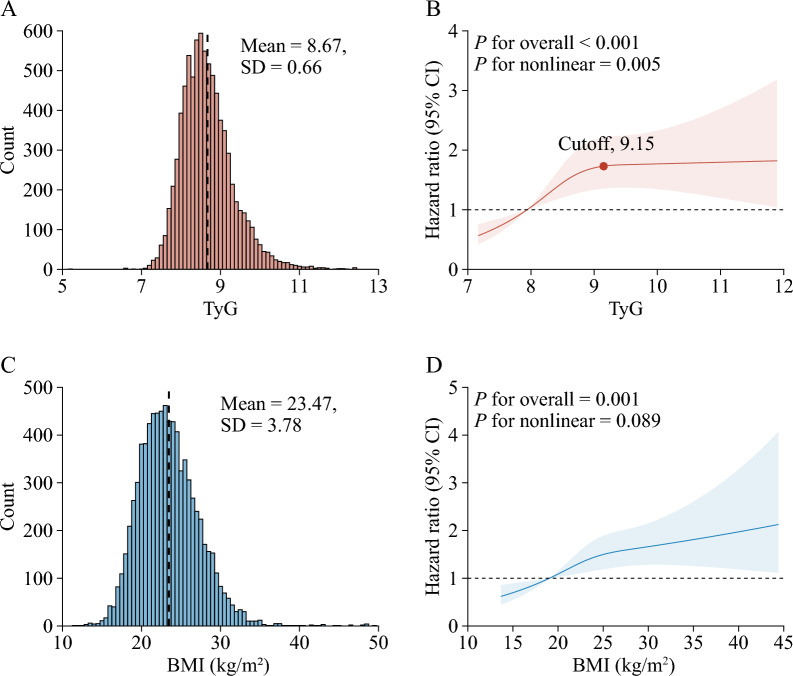
Nonlinear associations of triglyceride-glucose index and body mass index with stroke. **A**, **C** Distribution for TyG and BMI; **B**, **D** Graphs show HRs for stroke adjusted for age, gender, marital status, residence, education level, smoking status, and drinking status, hypertension, diabetes, heart disease, dyslipidaemia, kidney disease, and history of medication use for hypertension, diabetes, and dyslipidaemia. Data were fitted by Cox proportional hazards regression models. Solid lines indicate HRs, and shadow shapes indicate 95% CIs. Abbreviations: BMI, body mass index; CI, confidence interval; TyG, triglyceride-glucose

During a median follow-up of 7.1 years between 2011 and 2018, 585 participants experienced incident stroke, and the incidence rate of stroke was 7.1%. Figure 1 shows the association of BMI and TyG with incident stroke events. After adjusting for potential confounders (in model 3), by comparing BMI ≥ 28.0 kg/m^2^ with BMI < 24.0 kg/m^2^, the adjusted HRs were 1.38 (95% CI 1.08–1.77) for stroke events; by comparing quartile 4 of TyG with quartile 1, the adjusted HRs were 1.65 (95% CI 1.27–2.15) for stroke events. A nonlinear association between the TyG and risk of incident stroke events using RCS regression was found (for nonlinearity, *P* = 0.005; Fig. 2A, B). While a linear and positive association between the BMI and risk of incident stroke events was found (for nonlinearity, P = 0.089; Fig. 2C, D). Additional file 1: Fig. S3 and Table S3 in the Supplement showed a nonlinear association between the BMI and the TyG (for nonlinearity, *P* < 0.001).

Mediation analysis (in model 3) showed that an indirect association HR of 1.06 (95% CI 1.01–1.12) for BMI in 24.0–27.9 kg/m^2^ *vs* the reference BMI < 24.0 kg/m^2^, which increased to 1.11 (95% CI 1.01–1.22) for the BMI ≥ 28.0 kg/m^2^ group. Proportions mediated were 16.3% for BMI in 24.0–27.9 kg/m^2^ group and 53.8% for BMI ≥ 28.0 kg/m^2^ group (Table 2).

**Table 2:** Decomposition of the total association between BMI and the risk of stroke into direct and indirect associations mediated by the TyG

Model	Association		Proportion mediated
	Indirect HR (95% CI)	Direct HR (95% CI)	
Model 1 ^a^			
BMI < 24.0 kg/m^2^	1 [Reference]		
BMI in 24.0**–**27.9 kg/m^2^	1.06 (1.01**–**1.12)	1.67 (1.15**–**2.41)	10.5%
BMI ≥ 28.0 kg/m^2^	1.11 (1.01**–**1.21)	1.14 (1.08**–**1.22)	55.7%
Model 2 ^b^			
BMI < 24.0 kg/m^2^	1 [Reference]		
BMI in 24.0**–**27.9 kg/m^2^	1.06 (1.00**–**1.12)	1.74 (1.21**–**2.53)	9.7%
BMI ≥ 28.0 kg/m^2^	1.11 (1.01**–**1.21)	1.13 (1.08**–**1.18)	53.9%
Model 3 ^c^			
BMI < 24.0 kg/m^2^	1 [Reference]		
BMI in 24.0**–**27.9 kg/m^2^	1.06 (1.01**–**1.12)	1.37 (0.94**–**2.01)	16.3%
BMI ≥ 28.0 kg/m^2^	1.11 (1.01**–**1.22)	1.09 (0.62**–**1.93)	53.8%

BMI, body mass index; CI, confidence interval

^a^ Model 1 was adjusted for age, gender

^b^ Model 2 was adjusted for age, gender, marital status, residence, education level, smoking status, and drinking status

^c^ Model 3 was adjusted as model 2 plus hypertension, diabetes, heart disease, dyslipidaemia, kidney disease, and history of medication use for hypertension, diabetes, and dyslipidaemia

### Interaction and joint analyses of BMI and TyG with incident stroke

**Fig. 3:**
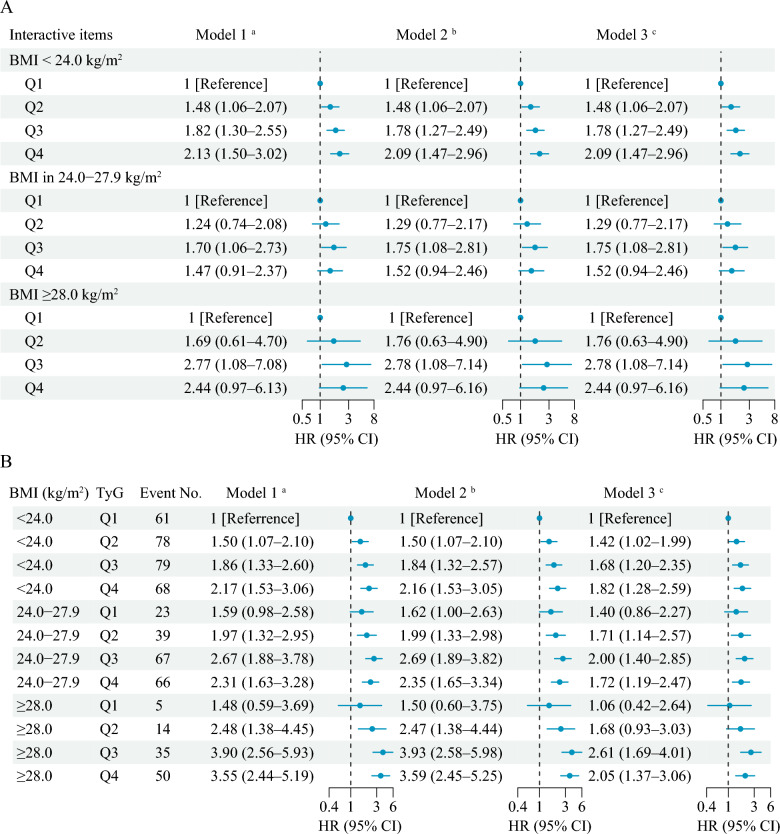
Interacting and joint effects of triglyceride-glucose index and body mass index on stroke risk. **A** Associations of triglyceride-glucose index with incident stroke by body mass index; **B** Joint associations of triglyceride-glucose index and body mass index with incident stroke. **a** Model 1 was adjusted for age, gender. **b** Model 2 was adjusted for age, gender, marital status, residence, education level, smoking status, and drinking status. **c** Model 3 was adjusted as model 2 plus hypertension, diabetes, heart disease, dyslipidaemia, kidney disease, and history of medication use for hypertension, diabetes, and dyslipidaemia. BMI, body mass index; CI, confidence interval; HR, hazard ratio; Q, quartile; TyG, triglyceride-glucose

No significant multiplicative and additive interactions were found between BMI and TyG on incident stroke (Additive: RERI = 1.78, 95% CI − 1.29–4.86; Multiplicative, HR = 1.40, 95% CI 0.86–2.27) (Table 3 and Fig. 3A). Figure 3B shows the joint association of BMI and TyG on the primary outcomes, and HRs for individuals of BMI ≥ 28.0 kg/m^2^ and quartile 4 of TyG compared with those with BMI < 24.0 kg/m^2^ and quartile 1 of TyG were 2.05 (95% CI 1.37–3.06) for incident stroke after adjusting for confounders.

**Table 3:** Interactive effects of triglyceride-glucose index and body mass index on incident stroke

Interactive items	Interactive effects (95% CI)		
	Model 1 ^a^	Model 2 ^b^	Model 3 ^c^
Additive effects			
RERI	3.40 (− 1.48–8.27)	3.44 (− 1.46–8.34)	1.78 (− 1.29–4.86)
AP	0.53 (0.25–0.81)	0.53 (0.26–0.81)	0.42 (0.07–0.76)
SI	2.68 (1.43–5.02)	2.72 (1.45–5.10)	2.19 (1.12–4.28)
Multiplicative effect	1.59 (0.98–2.58)	1.62 (1.00–2.63)	1.40 (0.86–2.27)

AP, proportion attributable to interaction; CI, confidence interval; RERI, relative excess risk due to interaction; SI, synergy index

^a^ Model 1 was adjusted for age, gender

^b^ Model 2 was adjusted for age, gender, marital status, residence, education level, smoking status, and drinking status

^c^ Model 3 was adjusted as model 2 plus hypertension, diabetes, heart disease, dyslipidaemia, kidney disease, and history of medication use for hypertension, diabetes, and dyslipidaemia

### Predictive value of BMI and TyG in incident stroke

**Fig. 4:**
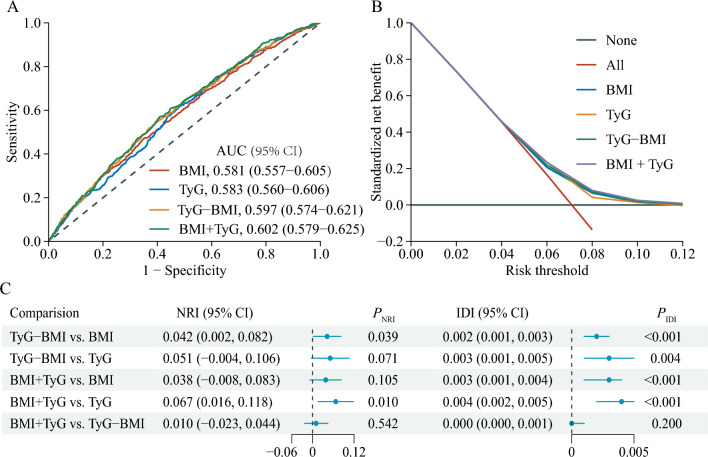
Predictive performance of the combined TyG and BMI for stroke. **A** The receiver operating characteristic (ROC) curve evaluating the discriminative capabilities by calculating the AUC; **B** Decision curve analysis to compare the clinical utility, the y-axis represents net benefits, calculated by subtracting the relative harm (false positives) from the benefits (true positives). The x-axis calculates the threshold probability; (**C**) NRI and IDI index for TyG combined with BMI. The TyG-BMI was calculated by the formula ln[TC (mg/dl) × FBG (mg/dl) / 2] × BMI (kg/m^2^). TyG + BMI indicates the combined effect of TyG and **BMI**. AUC, area under curve; BMI, body mass index; CI, confidence interval; TyG, triglyceride-glucose; NRI, net reclassification index; IDI, integrated discrimination improvement

We assessed the enhanced predictive value of combining BMI and TyG compared to their individual performance. The ROC curve indicated an AUC of 0.602 for the combined metric (Fig. 4A), and the decision curve confirmed its clinical relevance (Fig. 4B). Notably, the combined BMI + TyG index differed significantly from the individual BMI and TyG metrics (Fig. 4C). For instance, although the categorical NRI of 0.038 was not significant when comparing BMI + TyG to BMI (*P* = 0.105), the IDI of 0.003 was highly significant (*P* < 0.001). Similarly, against TyG alone, BMI + TyG yielded a significant NRI of 0.067 (*P* = 0.010) and IDI of 0.004 (*P* < 0.001). Finally, we evaluated whether includes BMI and TyG index would further increase the predictive value of traditional risk (Additional file 1): Table S4. The AUC by the traditional model significantly improve with the addition of BMI and TyG index (from 0.671 to 0.688, *P* < 0.001), the discriminatory power and risk reclassification also appeared to be substantially better, with the IDI of 0.003 (95% CI 0.001–0.005; *P* = 0.004), and the NRI of 0.182 (95% CI 0.098–0.266; *P* < 0.001). This suggests the superior efficacy of the combined index in this study.

### Subgroup and sensitivity analyses

The E-values showed the associations of BMI and TyG with stroke were robust (Additional file 1: Table S5). Additional file 1: Table S6-11 show results stratified by age, sex group. TyG mediates a higher proportion of BMI and stroke in participants ≥ 60 years compared with participants < 60 years (Additional file 1: Table S6); as well as, TyG mediates a higher proportion of BMI and stroke in females compared with males (Additional file 1: Table S7). Similar results were found when complete data analyses were conducted (Additional file 1: Table S12-14) or used TyG at Wave 3 (Additional file 1: Table S15-18). In addition, TyG significantly mediated 9.6% (95% CI 1.0–34.8%) of the BMI-associated elevated stroke risk when using the four-way decomposition method (Additional file 1: Table S19).

## Discussion

This nationwide prospective cohort study aimed to shed light on the mediating, interacting, and joint effects of two key variables, BMI and the TyG, in relation to stroke risk. Our findings have brought to the fore a nuanced perspective on how these factors intertwine and influence stroke risk assessment, offering important implications for clinical practice and public health strategies.

Our study reiterates the well-established association between elevated BMI and increased stroke risk, corroborating previous research findings [3, 4]. Obesity, as indicated by BMI, is recognized as a significant and modifiable risk factor for stroke [3, 4]. Particularly abdominal or visceral obesity, is associated with a host of metabolic abnormalities, including insulin resistance, dyslipidemia, and inflammation [27–29]. These metabolic disturbances contribute to the development and progression of atherosclerosis, a key contributor to stroke [30, 31]. Individuals who are overweight or obese are more likely to develop insulin resistance indicating early impaired glucose metabolism [32]. Studies have shown an association of insulin resistance with stroke independent of diabetes, which was even greater in the presence of obesity [10, 33, 34]. Therefore, insulin resistance might be a potentially important mediator of the association between BMI and the risk of stroke.

To our knowledge, our study is the first to examine the association between the TyG as a validated measure of insulin resistance and stroke risk and analyze its mediating role in BMI-related stroke risk. The TyG has been shown to be a simple measure of insulin resistance [35]. The TyG correlates with the euglycemic-hyperinsulinemic clamp test, and its validity is similar to the homeostatic model assessment insulin resistance index [36]. Owing to its easy availability and good performance, the TyG can be conveniently used in large-scale epidemiologic studies as a simple surrogate measure for insulin resistance. Our findings indicate that the TyG plays a partial mediating role in the link between BMI and stroke risk. The TyG, as an indicator of insulin resistance and metabolic health, provides a window into the complex metabolic processes through which elevated BMI may elevate stroke risk. Clinically, the identification of the TyG as a partial mediator in the BMI-stroke risk relationship underscores the importance of metabolic health assessment alongside BMI. Healthcare providers should consider evaluating insulin resistance and metabolic dysfunction in individuals with elevated BMI, as these factors may represent modifiable targets for stroke risk reduction. While TyG is a valuable and convenient tool for assessing insulin resistance, is not an officially accepted gold-standard measurement for insulin resistance. We will emphasize that it serves as a surrogate marker, which can provide insights into insulin sensitivity, but it may have limitations when compared to more direct and precise methods for measuring insulin resistance.

Our study also delved into the potentially interaction between BMI and the TyG in relation to stroke risk. In contrast to our initial hypothesis, our findings suggest that there is no significant synergy or interaction between these two factors in their impact on stroke risk. In essence, while both BMI and the TyG independently contribute to stroke risk [3, 4, 14, 15], their combined effect does not appear to exceed the sum of their individual effects. This observation introduces complexity into our understanding of how BMI and the TyG jointly influence stroke risk. Stroke, as a multifactorial condition, results from a multitude of interwoven risk factors, including hypertension, smoking, diet, genetics, and metabolic health [37–39]. The presence of these various factors may moderate the interaction between BMI and the TyG, potentially explaining the lack of a pronounced synergistic effect. Another possibility is that BMI and the TyG operate through distinct pathways in relation to stroke risk. While both factors are associated with metabolic dysfunction, their respective mechanisms may differ. Further research is needed to elucidate the specific pathways and mechanisms by which BMI and the TyG contribute to stroke risk.

While our study did not reveal a synergistic interaction between BMI and the TyG, it did underscore the importance of considering these factors jointly in stroke risk assessment. Our findings suggest that the combination of both BMI and the TyG may provide greater efficacy in predicting stroke risk than either factor alone. The concept of joint effects implies that individuals with elevated values in both BMI and the TyG may face a substantially higher risk of stroke compared to those with elevated values in only one of these variables. Notably, a more pronounced impact on stroke incidence was evident in individuals with a BMI ≥ 28.0 kg/m^2^ and falling within quartile 3 of TyG in comparison to those within quartile 4 of TyG. These findings suggest a potential nonlinearity in the joint effect of BMI and TyG on stroke. Such an observation aligns with the reasonable premise that disease risk is unlikely to exhibit a consistent increase with heightened exposure. However, the exact biological mechanism remains unknown.

This observation has practical implications for clinical practice and risk stratification. Identifying individuals at high risk based on the combined assessment of BMI and the TyG enables more targeted and tailored preventive strategies. Lifestyle modifications, including weight management, dietary adjustments, and physical activity, can be recommended with a heightened emphasis for those in the high-risk group. Additionally, healthcare providers may consider more aggressive management of cardiovascular risk factors, such as hypertension and dyslipidemia, in this population.

The clinical and public health implications of our findings underscore the importance of a comprehensive approach to stroke prevention. Because the epidemic of obesity is accompanied by a growing number of patients with stroke worldwide [40–42]. Obesity clearly is a modifiable risk factor and a considerable proportion of stroke and diabetes may be prevented if the general population maintained a normal BMI. Such as, bariatric surgery as a weight-reducing intervention was found to result in a significant reduction of insulin resistance [43, 44]. At the public health level, our study highlights the significance of addressing the obesity epidemic and promoting metabolic health. Public health initiatives aimed at reducing obesity rates and improving metabolic health have the potentially to make a substantial impact on lowering the overall burden of stroke. These efforts should encompass strategies to encourage healthy lifestyle behaviors, foster environments conducive to physical activity, and promote access to nutritious foods [45, 46].

Our findings are noteworthy as they derive from an extensive, representative cohort of the general Chinese population, monitored over an extended period. Such comprehensive tracking is essential for accurately examining longitudinal associations, especially those related to obesity, and their impact on stroke. Moreover, we employed a novel analytical tool rooted in the counterfactual framework. Unlike conventional methods for mediation analysis, this tool facilitates a mathematically coherent breakdown of the total association into direct and indirect associations, yielding transparent interpretations [47, 48]. Additionally, we computed the additive interactive effects of BMI and TyG on stroke. This approach offers greater utility for public health interventions than merely identifying deviations from multiplicativity when translating epidemiological findings.

However, our study has certain limitations that warrant consideration. First, we utilized BMI to identify overweight and obesity. While BMI is commonly employed and straightforward to compute, it does not provide an accurate representation of fat mass distribution and proportion. We did not have access to alternative metrics, such as waist circumference [49], waist-to-hip ratio [49, 50], or body fat composition analysis [51], which might offer a more precise assessment of visceral fat and serve as more sensitive indicators of stroke risk. Second, its observational design precludes the determination of causal links. Although our results suggest associations and interactions, establishing causality requires further investigations, encompassing randomized controlled trials and experimental designs. Third, as with many studies, stroke diagnosis in this research was based on self-reporting, posing a methodological challenge. The CHARLS dataset lacks medical records, making it impossible to validate self-reported stroke incidents. Nonetheless, it's pertinent to highlight that other large-scale studies, like the English Longitudinal Study of Ageing, have found a commendable concordance between self-reported strokes and medical documentation [52]. Fourth, we excluded participants who did not have blood samples or incomplete information on BMI and TyG, as excluded patients had higher BMI and TyG, which may underestimate the associations of TyG and BMI on stroke risk. Fifth, some confounding factors of the association between BMI and stroke, such as diet, physical activity, and family history of stroke were not adjusted in this study, but the E-values showed the associations were robust. Lastly, the BMI and the TyG were measured concurrently, raising the possibility of reverse causality. However, this concern was addressed through sensitivity analyses using TyG data from Wave 3, which yielded comparable results.

## Conclusions

Our findings indicate that the TyG serves as a valuable tool for identifying individuals at elevated risk of stroke. Furthermore, in our general population cohort, the TyG mediates more than 50% of the total association between BMI and stroke. Public health initiatives focused on weight reduction could potentially mitigate both insulin resistance and the overall burden of stroke.

### Supplementary Information

**Additional file 1:** Figure S1. Mediating pathway of the association of BMI with stroke. Figure S2. Flowchart of the study population. Figure S3. Nonlinear association between body mass index and triglyceride-glucose. Table S1. Baseline characteristics between participants included and not included. Table S2. Baseline characteristics of the include participants and those without blood samples and incomplete information on body mass index and triglyceride-glucose index. Table S3. Association between body mass index and triglyceride-glucose index using multiple linear regression. Table S4. Incremental predictive value of BMI and TyG index beyond traditional risk factors. Table S5. E-values of triglyceride-glucose index and body mass index with stroke. Table S6. Decomposition of the total association between BMI and the risk of stroke into direct and indirect associations mediated by the TyG index stratified by age. Table S7. Decomposition of the total association between BMI and the risk of stroke into direct and indirect associations mediated by the TyG index stratified by gender. Table S8. Interactive effects of triglyceride-glucose index and body mass index on incident stroke stratified by age. Table S9. Interactive effects of triglyceride-glucose index and body mass index on incident stroke stratified by gender. Table S10. Joint associations of triglyceride-glucose index and body mass index with incident stroke stratified by age. Table S11. Joint associations of triglyceride-glucose index and body mass index with incident stroke stratified by gender. Table S12. Decomposition of the total association between BMI and the risk of stroke into direct and indirect associations mediated by the TyG index in subpopulations of 7956 participants with complete data. Table S13. Interactive effects of triglyceride-glucose index and body mass index on incident stroke in subpopulations of 7 956 participants with complete data. Table S14. Joint associations of triglyceride-glucose index and body mass index with incident stroke in subpopulations of 7 956 participants with complete data. Table S15. Association between body mass index and triglyceride-glucose index at Wave 3 using multiple linear regression. Table S16. Decomposition of the total association between BMI and the risk of stroke into direct and indirect associations mediated by the TyG index at Wave 3. Table S17. Interactive effects of triglyceride-glucose index at Wave 3 and body mass index on incident stroke. Table S18. Joint associations of triglyceride-glucose index at Wave 3 and body mass index with incident stroke. Table S19. Mediated effects by TyG on the associations of ALP BMI with risk of stroke using the four-way decomposition method.

## Data Availability

Online repositories contain the datasets used in this investigation. The names of the repositories and accession numbers can be found at http://charls.pku.edu.cn/en.
