# Supplementary material for: Interacting and joint effects of triglyceride-glucose index (TyG) and body mass index on stroke risk and the mediating role of TyG in middle-aged and older Chinese adults: a nationwide prospective cohort study
Source: Cardiovasc Diabetol. 2024 Jan 13;23:30. doi: 10.1186/s12933-024-02122-4 (PMC10790273; doi:10.1186/s12933-024-02122-4)
Supplement: Supplementary file 1 — Additional file 1: Figure S1. Mediating pathway of the association of BMI with stroke. Figure S2. Flowchart of the study population. Figure S3. Nonlinear association between body mass index and triglyceride-glucose. Table S1. Baseline characteristics between participants included and not included. Table S2. Baseline characteristics of the include participants and those without blood samples and incomplete information on body mass index and triglyceride-glucose index. Table S3. Association between body mass index and triglyceride-glucose index using multiple linear regression. Table S4. Incremental predictive value of BMI and TyG index beyond traditional risk factors. Table S5. E-values of triglyceride-glucose index and body mass index with stroke. Table S6. Decomposition of the total association between BMI and the risk of stroke into direct and indirect associations mediated by the TyG index stratified by age. Table S7. Decomposition of the total association between BMI and the risk of stroke into direct and indirect associations mediated by the TyG index stratified by gender. Table S8. Interactive effects of triglyceride-glucose index and body mass index on incident stroke stratified by age. Table S9. Interactive effects of triglyceride-glucose index and body mass index on incident stroke stratified by gender. Table S10. Joint associations of triglyceride-glucose index and body mass index with incident stroke stratified by age. Table S11. Joint associations of triglyceride-glucose index and body mass index with incident stroke stratified by gender. Table S12. Decomposition of the total association between BMI and the risk of stroke into direct and indirect associations mediated by the TyG index in subpopulations of 7956 participants with complete data. Table S13. Interactive effects of triglyceride-glucose index and body mass index on incident stroke in subpopulations of 7 956 participants with complete data. Table S14. Joint associations of triglyceride-glucose i [file 12933_2024_2122_MOESM1_ESM.docx]

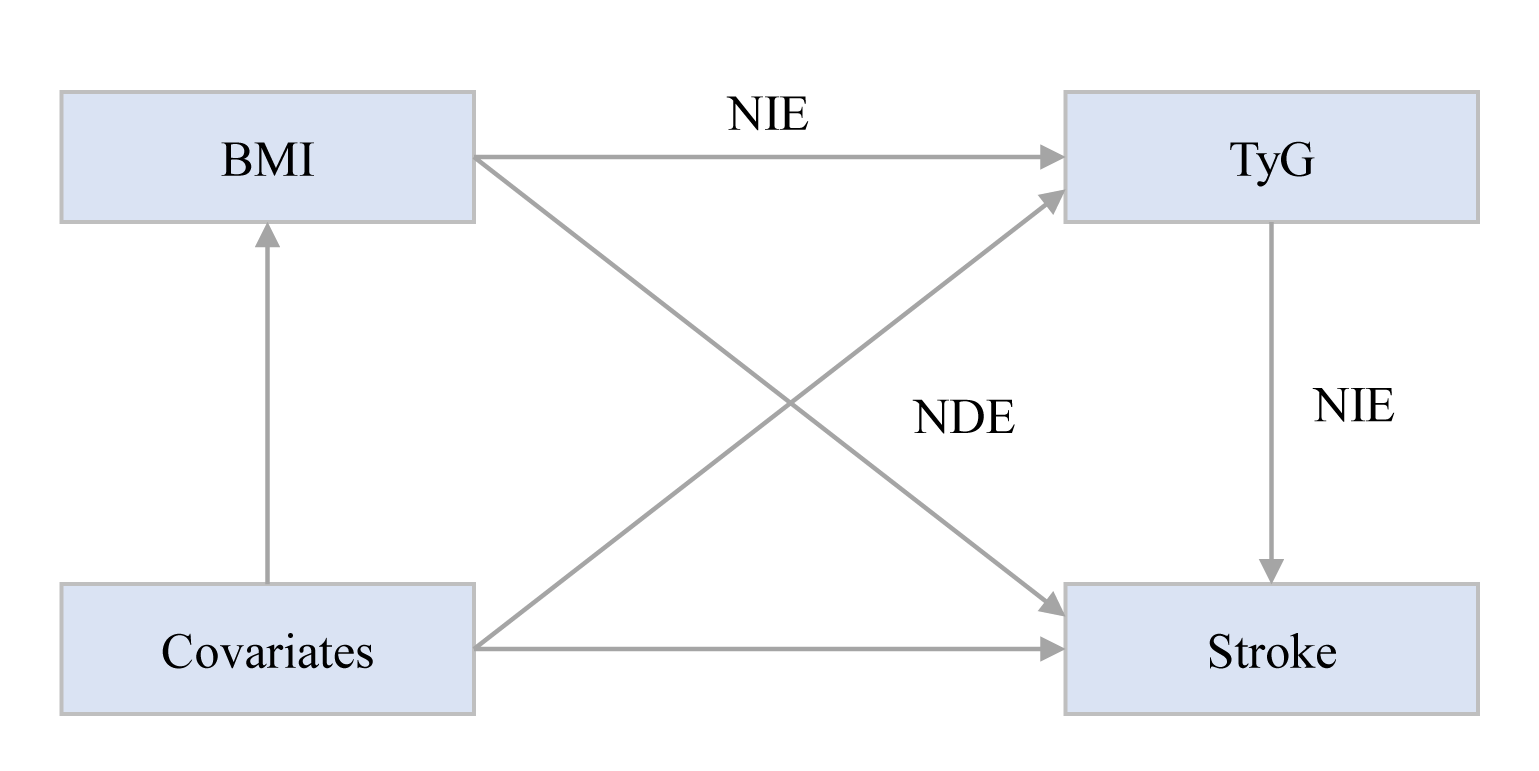


**Figure S1: Mediating pathway of the association of BMI with stroke.**

Direct acyclic graph of a structural model of mediation of the association between BMI and stroke by TyG. **Abbreviations**: BMI, body mass index; TyG, triglyceride-glucose; NDE indicates natural direct effects; NIE, natural indirect effects.


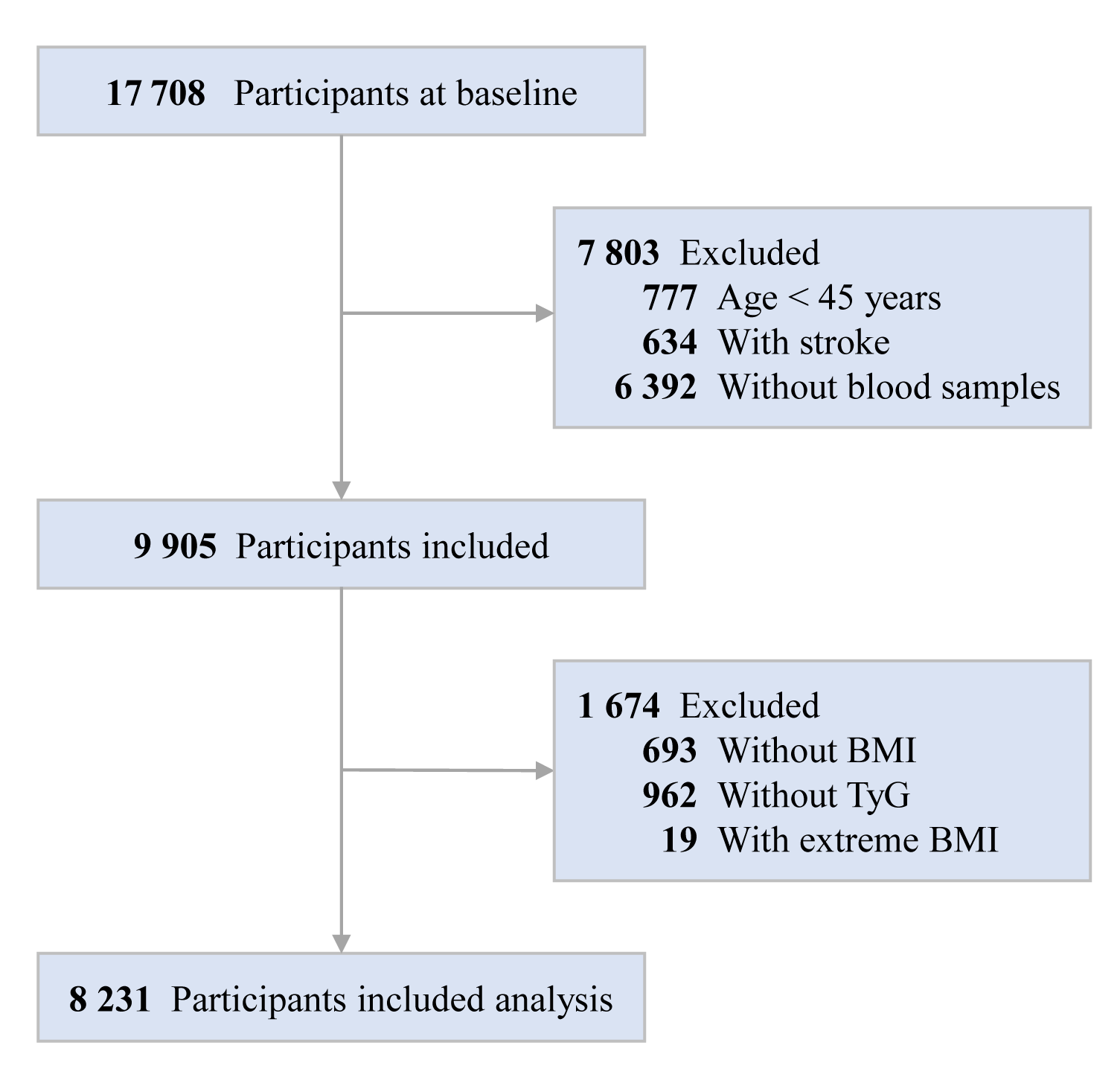


**Figure S2: Flowchart of the study population.**

**Abbreviations**: BMI: body mass index; TyG: triglyceride-glucose.


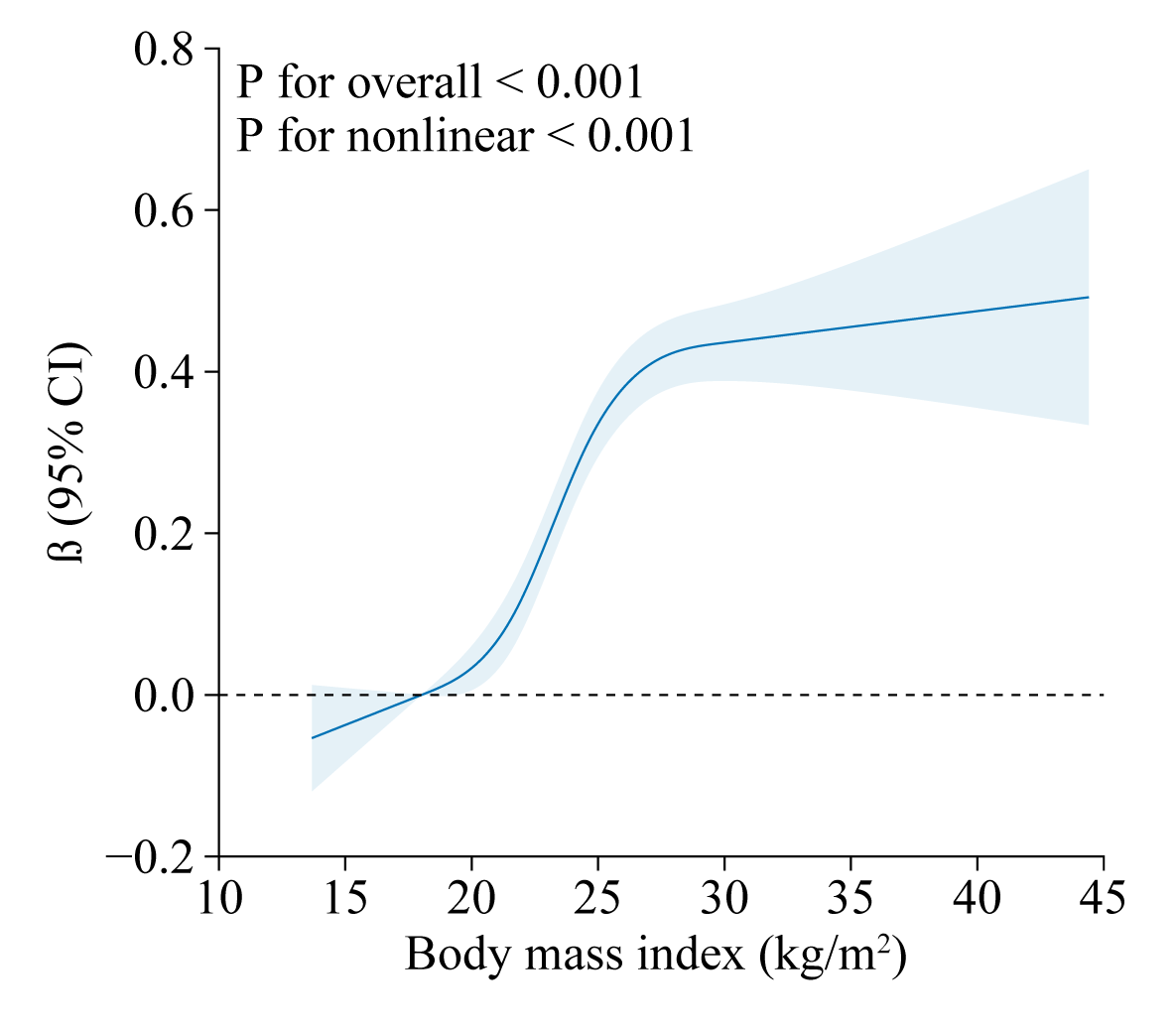


**Figure S3: Nonlinear association between body mass index and triglyceride-glucose.**

Graphs show β for triglyceride-glucose adjusted for age, gender, marital status, residence, education level, smoking status, and drinking status, hypertension, diabetes, heart disease, dyslipidaemia, kidney disease, and history of medication use for hypertension, diabetes, and dyslipidaemia. Data were fitted by a restricted cubic spline (RCS) linear regression model. Solid lines indicate β, and shadow shapes indicate 95% CIs.

Table S1: Baseline characteristics between participants included and not included

| Characteristic | Exclude (n=9477) | Include (n=8231) | *P* value ^a^ |
| --- | --- | --- | --- |
| Age, years | 57.84±10.83 | 59.23±9.32 | <0.001 |
| <60 | 5672 (59.9%) | 4571 (55.5%) |  |
| ≥60 | 3630 (38.3%) | 3660 (44.5%) |  |
| Missing | 175 (1.8%) | 0 (0.0%) |  |
| Gender |  |  | <0.001 |
| Male | 4663 (49.2%) | 3815 (46.3%) |  |
| Female | 4812 (50.8%) | 4416 (53.7%) |  |
| Missing | 2 (0.0%) | 0 (0.0%) |  |
| Marital status |  |  | <0.001 |
| Marred | 7313 (77.2%) | 6857 (83.3%) |  |
| Other | 2152 (22.7%) | 1374 (16.7%) |  |
| Missing | 12 (0.1%) | 0 (0.0%) |  |
| Residence |  |  | <0.001 |
| Urban | 4235 (44.7%) | 2936 (35.7%) |  |
| Rural | 5242 (55.3%) | 5295 (64.3%) |  |
| Education level |  |  | <0.001 |
| No formal education | 2390 (25.2%) | 2461 (29.9%) |  |
| Primary school | 3575 (37.7%) | 3345 (40.6%) |  |
| Middle or high school | 2928 (30.9%) | 2175 (26.4%) |  |
| College or above | 568 (6.0%) | 250 (3.0%) |  |
| Missing | 16 (0.2%) | 0 (0.0%) |  |
| Smoking status |  |  | <0.001 |
| Never | 5610 (59.2%) | 5009 (60.9%) |  |
| Former | 700 (7.4%) | 717 (8.7%) |  |
| Current | 2388 (25.2%) | 2483 (30.2%) |  |
| Missing | 779 (8.2%) | 22 (0.3%) |  |
| Drinking status |  |  | 0.691 |
| Never | 5515 (58.2%) | 4818 (58.5%) |  |
| Former | 759 (8.0%) | 684 (8.3%) |  |
| Current | 3042 (32.1%) | 2725 (33.1%) |  |
| Missing | 161 (1.7%) | 4 (0.0%) |  |
| Hypertension |  |  | 0.691 |
| No | 6884 (72.6%) | 6051 (73.5%) |  |
| Yes | 2398 (25.3%) | 2137 (26.0%) |  |
| Missing | 195 (2.1%) | 43 (0.5%) |  |
| Diabetes |  |  | 0.758 |
| No | 8699 (91.8%) | 7647 (92.9%) |  |
| Yes | 560 (5.9%) | 502 (6.1%) |  |
| Missing | 218 (2.3%) | 82 (1.0%) |  |
| Heart disease |  |  | 0.108 |
| No | 8104 (85.5%) | 7233 (87.9%) |  |
| Yes | 1165 (12.3%) | 965 (11.7%) |  |
| Missing | 208 (2.2%) | 33 (0.4%) |  |
| Dyslipidemia |  |  | 0.924 |
| No | 8252 (87.1%) | 7265 (88.3%) |  |
| Yes | 904 (9.5%) | 792 (9.6%) |  |
| Missing | 321 (3.4%) | 174 (2.1%) |  |
| Kidney disease |  |  | 0.119 |
| No | 8768 (92.5%) | 7705 (93.6%) |  |
| Yes | 496 (5.2%) | 483 (5.9%) |  |
| Missing | 213 (2.2%) | 43 (0.5%) |  |
| History of medication use for hypertension |  |  | 0.884 |
| No | 7456 (78.7%) | 6590 (80.1%) |  |
| Yes | 1817 (19.2%) | 1597 (19.4%) |  |
| Missing | 204 (2.2%) | 44 (0.5%) |  |
| History of medication use for diabetes |  |  | 0.428 |
| No | 8867 (93.6%) | 7825 (95.1%) |  |
| Yes | 389 (4.1%) | 323 (3.9%) |  |
| Missing | 221 (2.3%) | 83 (1.0%) |  |
| History of medication use for dyslipidemia |  |  | 0.725 |
| No | 8693 (91.7%) | 7643 (92.9%) |  |
| Yes | 455 (4.8%) | 410 (5.0%) |  |
| Missing | 329 (3.5%) | 178 (2.2%) |  |
| Systole blood pressure, mmHg |  |  | 0.062 |
| Mean±SD | 130.00±21.99 | 129.30±21.25 |  |
| Missing | 3888 | 80 |  |
| Diastolic blood pressure, mmHg |  |  | 0.001 |
| Mean±SD | 75.91±12.47 | 75.19±12.10 |  |
| Missing | 3888 | 81 |  |
| Body mass index, kg/m^2^ |  |  | 0.025 |
| Mean±SD | 25.20±56.67 | 23.47±3.78 |  |
| Missing | 4077 | 0 |  |
| FBG, mg/dl |  |  | 0.230 |
| Mean±SD | 110.98±40.75 | 110.02±35.82 |  |
| Missing | 6072 | 0 |  |
| TG, mg/dl |  |  | <0.001 |
| Mean±SD | 144.82±115.34 | 130.78±107.82 |  |
| Missing | 6052 | 0 |  |
| TyG |  |  | <0.001 |
| Mean±SD | 8.76±0.72 | 8.67±0.66 |  |
| Missing | 6072 | 0 |  |

Abbreviations: FBG, fasting blood glucose; TG, triglyceride; TyG, triglyceride-glucose.

^a^ *P* value was based on χ2 or analysis of variance or Kruskal-Wallis rank sum test where appropriate.

Table S2: Baseline characteristics of the include participants and those without blood samples and incomplete information on body mass index and triglyceride-glucose index

| Characteristic | Exclude (n=8066) | Include (n=8231) | *P* value ^a^ |
| --- | --- | --- | --- |
| Age, years | 58.67±10.09 | 59.23±9.32 | <0.001 |
| <60 | 4819 (59.7%) | 4571 (55.5%) |  |
| ≥60 | 3247 (40.3%) | 3660 (44.5%) |  |
| Gender |  |  | <0.001 |
| Male | 4125 (51.1%) | 3815 (46.3%) |  |
| Female | 3941 (48.9%) | 4416 (53.7%) |  |
| Marital status |  |  | <0.001 |
| Marred | 6230 (77.2%) | 6857 (83.3%) |  |
| Other | 1834 (22.7%) | 1374 (16.7%) |  |
| Missing | 2 (0.0%) | 0 (0.0%) |  |
| Residence |  |  | <0.001 |
| Urban | 3605 (44.7%) | 2936 (35.7%) |  |
| Rural | 4461 (55.3%) | 5295 (64.3%) |  |
| Education level |  |  | <0.001 |
| No formal education | 2014 (25.0%) | 2461 (29.9%) |  |
| Primary school | 2997 (37.2%) | 3345 (40.6%) |  |
| Middle or high school | 2549 (31.6%) | 2175 (26.4%) |  |
| College or above | 503 (6.2%) | 250 (3.0%) |  |
| Missing | 3 (0.0%) | 0 (0.0%) |  |
| Smoking status |  |  | 0.017 |
| Never | 4718 (58.5%) | 5009 (60.9%) |  |
| Former | 590 (7.3%) | 717 (8.7%) |  |
| Current | 2169 (26.9%) | 2483 (30.2%) |  |
| Missing | 589 (7.3%) | 22 (0.3%) |  |
| Drinking status |  |  | 0.404 |
| Never | 4668 (57.9%) | 4818 (58.5%) |  |
| Former | 638 (7.9%) | 684 (8.3%) |  |
| Current | 2733 (33.9%) | 2725 (33.1%) |  |
| Missing | 27 (0.3%) | 4 (0.0%) |  |
| Hypertension |  |  | 0.017 |
| No | 6060 (75.1%) | 6051 (73.5%) |  |
| Yes | 1963 (24.3%) | 2137 (26.0%) |  |
| Missing | 43 (0.5%) | 43 (0.5%) |  |
| Diabetes |  |  | 0.310 |
| No | 7545 (93.5%) | 7647 (92.9%) |  |
| Yes | 463 (5.7%) | 502 (6.1%) |  |
| Missing | 58 (0.7%) | 82 (1.0%) |  |
| Heart disease |  |  | 0.381 |
| No | 7048 (87.4%) | 7233 (87.9%) |  |
| Yes | 981 (12.2%) | 965 (11.7%) |  |
| Missing | 37 (0.5%) | 33 (0.4%) |  |
| Dyslipidemia |  |  | 0.241 |
| No | 7191 (89.2%) | 7265 (88.3%) |  |
| Yes | 736 (9.1%) | 792 (9.6%) |  |
| Missing | 139 (1.7%) | 174 (2.1%) |  |
| Kidney disease |  |  | 0.103 |
| No | 7597 (94.2%) | 7705 (93.6%) |  |
| Yes | 426 (5.3%) | 483 (5.9%) |  |
| Missing | 43 (0.5%) | 43 (0.5%) |  |
| History of medication use for hypertension |  |  | 0.064 |
| No | 6543 (81.1%) | 6590 (80.1%) |  |
| Yes | 1472 (18.2%) | 1597 (19.4%) |  |
| Missing | 51 (0.6%) | 44 (0.5%) |  |
| History of medication use for diabetes |  |  | 0.989 |
| No | 7688 (95.3%) | 7825 (95.1%) |  |
| Yes | 317 (3.9%) | 323 (3.9%) |  |
| Missing | 61 (0.8%) | 83 (1.0%) |  |
| History of medication use for dyslipidemia |  |  | 0.038 |
| No | 7573 (93.9%) | 7643 (92.9%) |  |
| Yes | 348 (4.3%) | 410 (5.0%) |  |
| Missing | 145 (1.8%) | 178 (2.2%) |  |
| Systole blood pressure, mmHg |  |  | 0.015 |
| Mean±SD | 130.26±21.86 | 129.30±21.25 |  |
| Missing | 3408 | 80 |  |
| Diastolic blood pressure, mmHg |  |  | 0.001 |
| Mean±SD | 75.91±12.40 | 75.19±12.10 |  |
| Missing | 3408 | 81 |  |
| Body mass index, kg/m^2^ |  |  | 0.034 |
| Mean±SD | 25.44±62.08 | 23.47±3.78 |  |
| Missing | 3570 | 0 |  |
| FBG, mg/dl |  |  | 0.098 |
| Mean±SD | 111.54±42.27 | 110.02±35.82 |  |
| Missing | 5471 | 0 |  |
| TG, mg/dl |  |  | <0.001 |
| Mean±SD | 145.43±113.41 | 130.78±107.82 |  |
| Missing | 5453 | 0 |  |
| TyG |  |  | <0.001 |
| Mean±SD | 8.76±0.73 | 8.67±0.66 |  |
| Missing | 5471 | 0 |  |

Abbreviations: FBG, fasting blood glucose; TG, triglyceride; TyG, triglyceride-glucose.

^a^ P value was based on χ^2^ or analysis of variance or Kruskal-Wallis rank sum test where appropriate.

Table S3: Association between body mass index and triglyceride-glucose index using multiple linear regression

| Body mass index | No. of total | Model 1 ^a^ | |  | Model 2 ^b^ | |  | Model 3 ^c^ | |
| --- | --- | --- | --- | --- | --- | --- | --- | --- | --- |
|  |  | β (95% CI) | P value |  | β (95% CI) | P value |  | β (95% CI) | P value |
| <24.0 | 4882 | 0 [Reference] |  |  | 0 [Reference] |  |  | 0 [Reference] |  |
| 24.0–27.9 | 2414 | 0.30 (0.27–0.33) | <0.001 |  | 0.28 (0.25–0.31) | <0.001 |  | 0.23 (0.20–0.26) | <0.001 |
| ≥28.0 | 935 | 0.49 (0.45–0.54) | <0.001 |  | 0.48 (0.43–0.52) | <0.001 |  | 0.36 (0.32–0.41) | <0.001 |

Abbreviations: CI, confidence interval.

^a^ Adjusted for age, gender.

^b^ Adjusted for age, gender, marital status, residence, education level, smoking status, and drinking status.

^c^ Adjusted for age, gender, marital status, residence, education level, smoking status, drinking status, hypertension, diabetes, heart disease, dyslipidaemia, kidney disease, and history of medication use for hypertension, diabetes, and dyslipidaemia.

**Table S4: Incremental predictive value of BMI and TyG index beyond traditional risk factors.**

| Models | AUC (95% CI) | *P*_AUC_ | NRI (95% CI) | *P*_NRI_ | IDI (95% CI) | *P*_IDI_ |
| --- | --- | --- | --- | --- | --- | --- |
| Traditional model ^a^ | 0.671 (0.648–0.695) |  | Reference |  | Reference |  |
| Traditional model + BMI + TyG | 0.688 (0.667–0.710) | <0.001 | 0.182 (0.098, 0.266) | <0.001 | 0.003 (0.001, 0.005) | 0.004 |

**Abbreviations:** AUC, area under curve; BMI, body mass index; CI, confidence interval; TyG, triglyceride-glucose; NRI, net reclassification index; IDI, integrated discrimination improvement.

^a^ Traditional model based on age, gender, marital status, residence, education level, smoking status, drinking status, hypertension, diabetes, heart disease, dyslipidaemia, kidney disease, and history of medication use for hypertension, diabetes, and dyslipidaemia.

**Table S5: E-values of triglyceride-glucose index and body mass index with stroke**

| Variable | Evalue (95% CI) ^a^ |
| --- | --- |
| TyG |  |
| Q1 [5.18, 8.22] | Reference |
| Q2 (8.22, 8.59] | 2.14 (1.34–3.06) |
| Q3 (8.59, 9.02] | 2.90 (2.04–3.97) |
| Q4 (9.02, 13.00] | 2.69 (1.86–3.72) |
| BMI (kg/m^2^) |  |
| <24.0 | Reference |
| 24.0–27.9 | 1.71 (1.00–2.28) |
| ≥28.0 | 2.10 (1.37–2.94) |

Abbreviations: BMI, body mass index; CI, confidence interval; Q, quartile; TyG, triglyceride-glucose.

^a^ Adjusted for age, gender, marital status, residence, education level, smoking status, drinking status, hypertension, diabetes, heart disease, dyslipidaemia, kidney disease, and history of medication use for hypertension, diabetes, and dyslipidaemia.

Table S6: Decomposition of the total association between BMI and the risk of stroke into direct and indirect associations mediated by the TyG index stratified by age

| Subgroub | Association | | Proportion mediated |
| --- | --- | --- | --- |
|  | Indirect HR (95% CI) | Direct HR (95% CI) |  |
| Age < 60 years |  |  |  |
| BMI < 24.0 kg/m^2^ | 1 [Reference] |  |  |
| BMI in 24.0–27.9 kg/m^2^ | 1.08 (0.99–1.17) | 1.84 (1.02–3.33) | 10.9% |
| BMI ≥28.0 kg/m^2^ | 1.16 (1.00–1.35) | 1.58 (0.69–3.61) | 24.4% |
| Age ≥ 60 years |  |  |  |
| BMI < 24.0 kg/m^2^ | 1 [Reference] |  |  |
| BMI in 24.0–27.9 kg/m^2^ | 1.05 (0.97–1.14) | 1.10 (0.66–1.84) | 33.7% |
| BMI ≥28.0 kg/m^2^ | 1.07 (0.96–1.18) | 1.19 (0.37–1.87) | 56.3% |

Abbreviations: BMI, body mass index; CI, confidence interval; HR, hazard ratio.

All models were adjusted for gender, marital status, residence, education level, smoking status, and drinking status, hypertension, diabetes, heart disease, dyslipidaemia, kidney disease, and history of medication use for hypertension, diabetes, and dyslipidaemia.

Table S7: Decomposition of the total association between BMI and the risk of stroke into direct and indirect associations mediated by the TyG index stratified by gender

| Subgroub | Association | | Proportion mediated |
| --- | --- | --- | --- |
|  | Indirect HR (95% CI) | Direct HR (95% CI) |  |
| Male |  |  |  |
| BMI < 24.0 kg/m^2^ | 1 [Reference] |  |  |
| BMI in 24.0–27.9 kg/m^2^ | 1.05 (0.97–1.14) | 1.47 (0.89–2.42) | 12.1% |
| BMI ≥28.0 kg/m^2^ | 1.10 (0.96–1.23) | 1.17 (0.53–2.59) | 38.3% |
| Female |  |  |  |
| BMI < 24.0 kg/m^2^ | 1 [Reference] |  |  |
| BMI in 24.0–27.9 kg/m^2^ | 1.08 (1.00–1.16) | 1.21 (0.67–2.19) | 47.1% |
| BMI ≥28.0 kg/m^2^ | 1.13 (1.00–1.28) | 1.07 (0.48–2.39) | 51.4% |

Abbreviations: BMI, body mass index; CI, confidence interval; HR, hazard ratio.

All models were adjusted for age, marital status, residence, education level, smoking status, and drinking status, hypertension, diabetes, heart disease, dyslipidaemia, kidney disease, and history of medication use for hypertension, diabetes, and dyslipidaemia.

Table S8: Interactive effects of triglyceride-glucose index and body mass index on incident stroke stratified by age

| Interactive items | Interactive effect (95% CI) ^a^ | |
| --- | --- | --- |
|  | Age < 60 years | Age ≥ 60 years |
| Additive effects |  |  |
| RERI | 0.55 (-1.68–2.78) | 3.56 (-3.36–10.47) |
| AP | 0.23 (-0.40–0.85) | 0.54 (0.16–0.92) |
| SI | 1.64 (0.49–5.41) | 2.75 (1.14–6.65) |
| Multiplicative effect | 1.18 (0.59–2.34) | 1.64 (0.83–3.25) |

Abbreviations: AP, proportion attributable to interaction; CI, confidence interval; RERI, relative excess risk due to interaction; SI, synergy index.

^a^ All models were adjusted for gender, marital status, residence, education level, smoking status, and drinking status, hypertension, diabetes, heart disease, dyslipidaemia, kidney disease, and history of medication use for hypertension, diabetes, and dyslipidaemia.

Table S9: Interactive effects of triglyceride-glucose index and body mass index on incident stroke stratified by gender

| Interactive items | Interactive effect (95% CI) ^a^ | |
| --- | --- | --- |
|  | Male | Female |
| Additive effects |  |  |
| RERI | 0.98 (-2.31–4.27) | 2.50 (-3.22–8.21) |
| AP | 0.28 (-0.32–0.89) | 0.50 (0.06–0.93) |
| SI | 1.66 (0.55–5.03) | 2.65 (1.09–6.46) |
| Multiplicative effect | 1.17 (0.56–2.44) | 1.63 (0.83–3.20) |

Abbreviations: AP, proportion attributable to interaction; CI, confidence interval; RERI, relative excess risk due to interaction; SI, synergy index.

^a^ All models were adjusted for age, marital status, residence, education level, smoking status, and drinking status, hypertension, diabetes, heart disease, dyslipidaemia, kidney disease, and history of medication use for hypertension, diabetes, and dyslipidaemia.

Table S10: Joint associations of triglyceride-glucose index and body mass index with incident stroke stratified by age

| BMI (kg/m^2^) | TyG | HR (95% CI) ^a^ | |
| --- | --- | --- | --- |
|  |  | Age < 60 years | Age ≥ 60 years |
| <24.0 | Q1 | 1 [Reference] | 1 [Reference] |
| <24.0 | Q2 | 0.85 (0.48–1.51) | 1.86 (1.21–2.85) |
| <24.0 | Q3 | 1.51 (0.89–2.54) | 1.81 (1.17–2.82) |
| <24.0 | Q4 | 1.35 (0.78–2.36) | 2.22 (1.40–3.51) |
| 24.0–27.9 | Q1 | 1.18 (0.59–2.34) | 1.64 (0.83–3.25) |
| 24.0–27.9 | Q2 | 1.98 (1.14–3.46) | 1.39 (0.75–2.57) |
| 24.0–27.9 | Q3 | 1.76 (1.04–2.99) | 2.14 (1.32–3.49) |
| 24.0–27.9 | Q4 | 1.60 (0.95–2.71) | 1.71 (1.03–2.86) |
| ≥28.0 | Q1 | 1.13 (0.34–3.77) | 0.96 (0.23–4.04) |
| ≥28.0 | Q2 | 1.57 (0.70–3.51) | 1.86 (0.77–4.50) |
| ≥28.0 | Q3 | 3.04 (1.68–5.51) | 2.24 (1.18–4.25) |
| ≥28.0 | Q4 | 1.59 (0.90–2.79) | 2.42 (1.34–4.35) |

Abbreviations: BMI, body mass index; CI, confidence interval; HR, hazard ratio; Q, quartile; TyG, triglyceride-glucose.

^a^ All models were adjusted for gender, marital status, residence, education level, smoking status, and drinking status, hypertension, diabetes, heart disease, dyslipidaemia, kidney disease, and history of medication use for hypertension, diabetes, and dyslipidaemia.

Table S11: Joint associations of triglyceride-glucose index and body mass index with incident stroke stratified by gender

| BMI (kg/m^2^) | TyG | HR (95% CI) ^a^ | |
| --- | --- | --- | --- |
|  |  | Male | Female |
| <24.0 | Q1 | 1 [Reference] | 1 [Reference] |
| <24.0 | Q2 | 1.38 (0.89–2.14) | 1.42 (0.84–2.41) |
| <24.0 | Q3 | 1.46 (0.92–2.31) | 1.90 (1.14–3.17) |
| <24.0 | Q4 | 2.04 (1.29–3.22) | 1.62 (0.93–2.81) |
| 24.0–27.9 | Q1 | 1.17 (0.56–2.44) | 1.63 (0.83–3.20) |
| 24.0–27.9 | Q2 | 1.52 (0.85–2.72) | 1.90 (1.05–3.44) |
| 24.0–27.9 | Q3 | 2.43 (1.51–3.91) | 1.69 (0.97–2.94) |
| 24.0–27.9 | Q4 | 1.77 (1.07–2.94) | 1.56 (0.91–2.70) |
| ≥28.0 | Q1 | 1.19 (0.36–3.91) | 0.92 (0.22–3.93) |
| ≥28.0 | Q2 | 1.43 (0.55–3.69) | 1.84 (0.84–4.03) |
| ≥28.0 | Q3 | 2.88 (1.45–5.72) | 2.56 (1.41–4.64) |
| ≥28.0 | Q4 | 2.53 (1.44–4.45) | 1.68 (0.93–3.05) |

Abbreviations: BMI, body mass index; CI, confidence interval; HR, hazard ratio; Q, quartile; TyG, triglyceride-glucose.

^a^ All models were adjusted for age, marital status, residence, education level, smoking status, and drinking status, hypertension, diabetes, heart disease, dyslipidaemia, kidney disease, and history of medication use for hypertension, diabetes, and dyslipidaemia.

Table S12: Decomposition of the total association between BMI and the risk of stroke into direct and indirect associations mediated by the TyG index in subpopulations of 7956 participants with complete data

| Subgroub | Association | | Proportion mediated |
| --- | --- | --- | --- |
|  | Indirect HR (95% CI) | Direct HR (95% CI) |  |
| BMI < 24.0 kg/m^2^ | 1 [Reference] |  |  |
| BMI in 24.0–27.9 kg/m^2^ | 1.06 (1.00-1.12) | 1.34 (0.92-1.97) | 17.2% |
| BMI ≥28.0 kg/m^2^ | 1.11 (1.01-1.22) | 1.07 (0.60-1.87) | 62.6% |

Abbreviations: BMI, body mass index; CI, confidence interval; HR, hazard ratio.

All models were adjusted for age, gender, marital status, residence, education level, smoking status, and drinking status, hypertension, diabetes, heart disease, dyslipidaemia, kidney disease, and history of medication use for hypertension, diabetes, and dyslipidaemia.

Table S13: Interactive effects of triglyceride-glucose index and body mass index on incident stroke in subpopulations of 7 956 participants with complete data

| Interactive items | Interactive effect (95% CI) ^a^ |
| --- | --- |
| Additive effects |  |
| RERI | 2.34 (-3.05–7.72) |
| AP | 0.49 (0.05–0.93) |
| SI | 2.65 (1.09–6.42) |
| Multiplicative effect | 1.63 (0.83–3.20) |

Abbreviations: AP, proportion attributable to interaction; CI, confidence interval; RERI, relative excess risk due to interaction; SI, synergy index.

^a^ All models were adjusted for age, gender, marital status, residence, education level, smoking status, and drinking status, hypertension, diabetes, heart disease, dyslipidaemia, kidney disease, and history of medication use for hypertension, diabetes, and dyslipidaemia.

Table S14: Joint associations of triglyceride-glucose index and body mass index with incident stroke in subpopulations of 7 956 participants with complete data

| BMI (kg/m^2^) | TyG | HR (95% CI) ^a^ |
| --- | --- | --- |
| <24.0 | Q1 | 1 [Reference] |
| <24.0 | Q2 | 1.41 (0.83–2.40) |
| <24.0 | Q3 | 1.85 (1.11–3.09) |
| <24.0 | Q4 | 1.62 (0.93–2.81) |
| 24.0–27.9 | Q1 | 1.64 (0.84–3.22) |
| 24.0–27.9 | Q2 | 1.87 (1.03–3.38) |
| 24.0–27.9 | Q3 | 1.67 (0.95–2.92) |
| 24.0–27.9 | Q4 | 1.51 (0.87–2.62) |
| ≥28.0 | Q1 | 0.94 (0.22–4.01) |
| ≥28.0 | Q2 | 1.90 (0.87–4.16) |
| ≥28.0 | Q3 | 2.44 (1.33–4.48) |
| ≥28.0 | Q4 | 1.71 (0.94–3.10) |

Abbreviations: BMI, body mass index; CI, confidence interval; HR, hazard ratio; Q, quartile; TyG, triglyceride-glucose.

^a^ All models were adjusted for age, gender, marital status, residence, education level, smoking status, and drinking status, hypertension, diabetes, heart disease, dyslipidaemia, kidney disease, and history of medication use for hypertension, diabetes, and dyslipidaemia.

Table S15: Association between body mass index and triglyceride-glucose index at Wave 3 using multiple linear regression

| Body mass index | No. of total | Model 1 ^a^ | |  | Model 2 ^b^ | |  | Model 3 ^c^ | |
| --- | --- | --- | --- | --- | --- | --- | --- | --- | --- |
|  |  | β (95% CI) | P value |  | β (95% CI) | P value |  | β (95% CI) | P value |
| <24.0 | 3280 | 0 [Reference] |  |  | 0 [Reference] |  |  | 0 [Reference] |  |
| 24.0–27.9 | 1643 | 0.32 (0.28–0.35) | <0.001 |  | 0.30 (0.26–0.34) | <0.001 |  | 0.26 (0.22–0.30) | <0.001 |
| ≥28.0 | 671 | 0.46 (0.41–0.51) | <0.001 |  | 0.44 (0.39–0.49) | <0.001 |  | 0.35 (0.29–0.40) | <0.001 |

Abbreviations: CI, confidence interval.

^a^ Adjusted for age, gender.

^b^ Adjusted for age, gender, marital status, residence, education level, smoking status, and drinking status.

^c^ Adjusted for age, gender, marital status, residence, education level, smoking status, drinking status, hypertension, diabetes, heart disease, dyslipidaemia, kidney disease, and history of medication use for hypertension, diabetes, and dyslipidaemia.

Table S16: Decomposition of the total association between BMI and the risk of stroke into direct and indirect associations mediated by the TyG index at Wave 3

| Subgroub | Association | | Proportion mediated |
| --- | --- | --- | --- |
|  | Indirect HR (95% CI) | Direct HR (95% CI) |  |
| BMI < 24.0 kg/m^2^ | 1 [Reference] |  |  |
| BMI in 24.0–27.9 kg/m^2^ | 1.03 (0.96–1.11) | 1.46 (0.89–2.39) | 8.0% |
| BMI ≥28.0 kg/m^2^ | 1.05 (0.95–1.17) | 1.06 (0.54–2.09) | 45.1% |

Abbreviations: BMI, body mass index; CI, confidence interval; HR, hazard ratio.

All models were adjusted for age, gender, marital status, residence, education level, smoking status, and drinking status, hypertension, diabetes, heart disease, dyslipidaemia, kidney disease, TyG at baseline, and history of medication use for hypertension, diabetes, and dyslipidaemia.

Table S17: Interactive effects of triglyceride-glucose index at Wave 3 and body mass index on incident stroke

| Interactive items | Interactive effect (95% CI) ^a^ |
| --- | --- |
| Additive effects |  |
| RERI | 0.64 (-1.36–2.65) |
| AP | 0.24 (-0.27–0.74) |
| SI | 1.59 (0.62–4.08) |
| Multiplicative effect | 1.15 (0.65–2.02) |

Abbreviations: AP, proportion attributable to interaction; CI, confidence interval; RERI, relative excess risk due to interaction; SI, synergy index.

^a^ All models were adjusted for age, gender, marital status, residence, education level, smoking status, and drinking status, hypertension, diabetes, heart disease, dyslipidaemia, kidney disease, TyG at baseline, and history of medication use for hypertension, diabetes, and dyslipidaemia.

Table S18: Joint associations of triglyceride-glucose index at Wave 3 and body mass index with incident stroke

| BMI (kg/m^2^) | TyG at Wave 3 | HR (95% CI) ^a^ |
| --- | --- | --- |
| <24.0 | Q1 | 1 [Reference] |
| <24.0 | Q2 | 2.45 (1.30–4.61) |
| <24.0 | Q3 | 2.12 (1.11–4.03) |
| <24.0 | Q4 | 1.87 (0.95–3.68) |
| 24.0–27.9 | Q1 | 2.27 (1.02–5.03) |
| 24.0–27.9 | Q2 | 2.15 (1.04–4.42) |
| 24.0–27.9 | Q3 | 1.82 (0.90–3.66) |
| 24.0–27.9 | Q4 | 2.12 (1.10–4.08) |
| ≥28.0 | Q1 | 2.51 (0.89–7.09) |
| ≥28.0 | Q2 | 1.80 (0.68–4.76) |
| ≥28.0 | Q3 | 3.52 (1.73–7.14) |
| ≥28.0 | Q4 | 1.97 (0.96–4.06) |

Abbreviations: BMI, body mass index; CI, confidence interval; HR, hazard ratio; Q, quartile; TyG, triglyceride-glucose.

^a^ All models were adjusted for age, gender, marital status, residence, education level, smoking status, and drinking status, hypertension, diabetes, heart disease, dyslipidaemia, kidney disease, TyG at baseline, and history of medication use for hypertension, diabetes, and dyslipidaemia.

**Table S19: Mediated effects by TyG on the associations of ALP BMI with risk of stroke using the four-way decomposition method**

| Component ^a^ | Excess relative hazard  (95% CI) ^b^ | Proportion attributable  (95% CI) ^b^ |
| --- | --- | --- |
| CDE | 0.480 (0.272 to 0.783) | 94.1% (71.7% to 119.1%) |
| INT_ref_ | -0.009 (-0.085 to 0.055) | -1.7% (-18.4% to 11.8%) |
| INT_med_ | -0.010 (-0.113 to 0.073) | -1.9% (-23.6% to 12.9%) |
| PIE | 0.049 (0.001 to 0.144) | 9.6% (1.0% to 34.8%) |

Abbreviations: BMI, body mass index; CDE, controlled direct effect; INT_ref_, reference interaction; INT_med_, mediated interaction; PIE, pure indirect effect.

^a^ As an alternative mediation decomposition, the CDE (due neither to mediation nor interaction) and the INT_ref_ (due to interaction only) combine to give the pure direct effect (PDE); PDE and the INT_med_ (due to mediation and interaction) combine to give the total direct effect (TDE); and TDE and the PIE (due to mediation only) combine to give the total effect (TE).

^b^ Two models were constructed: one involved a multivariable logistic regression model for TyG (mediator, ≥9.15 *vs* <9.15), conditioned on BMI (exposure, ≥24.0 kg/m^2^ *vs* <24.0 kg/m^2^), while the other involved a multivariable Cox proportional hazard regression model for stroke (outcome, time-to-event data), conditioned on BMI and TyG. All models were adjusted for age, gender, marital status, residence, education level, smoking status, and drinking status, hypertension, diabetes, heart disease, dyslipidaemia, kidney disease, and history of medication use for hypertension, diabetes, and dyslipidaemia. The the effect was statistically significant when its CI did not include 0, and the 95% CI was calculated by a bootstrap resampling method by resampling 1 000 bootstrap.
